# Human sleeping pose estimation from IR images for in-bed patient monitoring using image processing and deep learning techniques

**DOI:** 10.1016/j.heliyon.2024.e36823

**Published:** 2024-08-25

**Authors:** Shahriar Kabir Nahin, Sanjay Acharjee, Sawradip Saha, Aurick Das, Shahruk Hossain, Mohammad Ariful Haque

**Affiliations:** Department of Electrical and Electronic Engineering, Bangladesh University of Engineering and Technology, Dhaka, 1205, Bangladesh

**Keywords:** DNN, Domain adaptation, IR image, Pose estimation

## Abstract

Human Pose Estimation (HPE) is a crucial step towards understanding people in images and videos. HPE provides geometric and motion information of the human body, which has been applied to a wide range of applications (e.g., human-computer interaction, motion analysis, augmented reality, virtual reality, healthcare, etc.). An extremely useful task of this kind is the 2D pose estimation of bedridden patients from infrared (IR) images. Here, the IR imaging modality is preferred due to privacy concerns and the need for monitoring both uncovered and covered patients at different levels of illumination. The major drawback of this research problem is the unavailability of covered examples, which are very costly to collect and time-consuming to label. In this work, a deep learning-based framework was developed for human sleeping pose estimation on covered images using only the uncovered training images. In the training scheme, two different image augmentation techniques, a statistical approach as well as a GAN-based approach, were explored for domain adaptation, where the statistical approach performed better. The accuracy of the model trained on the statistically augmented dataset was improved by 124 % as compared with the model trained on non-augmented images. To handle the scarcity of training infrared images, a transfer learning strategy was used by pre-training the model on an RGB pose estimation dataset, resulting in a further increment in accuracy of 4 %. Semi-supervised learning techniques, with a novel pose discriminator model in the loop, were adopted to utilize the unannotated training data, resulting in a further 3 % increase in accuracy. Thus, significant improvement has been shown in the case of 2D pose estimation from infrared images, with a comparatively small amount of annotated data and a large amount of unannotated data by using the proposed training pipeline powered by heavy augmentation.

## 1 Introduction

Human Pose Estimation (HPE) is a foundational problem in computer vision with applications including pedestrian intent recognition for self-driving cars [1] to patient gait and posture monitoring [2], and animating 3D characters [3]. The medical field has particularly benefited from HPE, enabling continuous monitoring in home and hospital settings [4]. Applications include assistive systems [5], infant pose estimation [6], rehabilitation [7], and patient-specific pose estimation [8].

Recently, in-bed human pose estimation has gained attention on monitoring individual's positions while lying or sleeping. This is useful for diagnosing sleep disorders, evaluating sleep quality, and tracking position changes to aid post-surgery recovery [9]. This task is challenging due to limited data, privacy concerns, and varying illumination. Traditional methods using RGB images are limited and dependent on visible light [10]. Long-wavelength infrared (LWIR) images are preferred for their ability to capture body heat and penetrate bed coverings, making them suitable for low-light conditions.

Despite advancements in HPE, little work has focused on key-point detection and pose estimation using LWIR images [11,12]. The lack of sufficient annotated LWIR data hinders the training of supervised deep learning models for accurate pose estimation in real-world settings. Furthermore, annotation becomes complicated when patients are covered by thick blankets [13]. Estimating human poses from covered images using uncovered data samples can be viewed as a domain adaptation problem. Although domain adaptation is well-reported in the literature, mainstream algorithms primarily focus on classification rather than regression tasks like pose estimation [14].

Inspired by the IEEE Video and Image Processing Cup 2021, we propose a deep learning framework for in-bed HPE. We use a subset of the Simultaneously Collected Multi-Modal Lying Pose (SLP) dataset [15], focusing on LWIR images. While other datasets like Microsoft COCO [16], MPII [17], and Leeds Sports [18] contain RGB images, they do not specifically address in-bed poses. Our approach includes a two-staged training pipeline with statistical and GAN-based augmentation techniques. Transfer learning from the MPII RGB pose estimation dataset and semi-supervised learning with unannotated data are employed. During inference, predictions from original and flipped images are averaged for improved accuracy, followed by post-processing to extract key points.

The key contributions of this paper are.

• Addressing domain mismatch through comprehensive statistical techniques and GAN-based augmentation.
• Mitigating data scarcity through transfer learning and an innovative semi-supervised learning approach utilizing a pose discriminator model.

## 2 Related work

HPE has been an active research area of computer vision, which has seen incremental improvements, particularly following the emergence of deep learning in 2012. In recent years, HPE has been a popular research area to identify unsafe behaviors using data processing algorithms, such as target detection, feature extraction, and feature analysis [[19], [20], [21]]. Deep learning-based pose estimation techniques are broadly categorized into regression-based and detection-based approaches. Regression-based approaches directly predict the coordinates of human body key-points from the input image features [22], while detection-based methods generate heatmaps for each key-point and determine the location by the peak in the heatmap [23]. The *Deeppose* [22] paper was one of the first to integrate deep learning with 2D pose estimation using the regression approach. Subsequent methods demonstrated that predicting heatmaps for individual key-points generally outperforms direct key-point regression [23].

The space of 2D human body poses is highly structured because of certain body part proportions, physical connectivity, joint motion limitations, and left-right symmetries, [[24], [25], [26]]. Performance was significantly improved when these constraints were utilized. Convolutional Pose Machines [27] a sequential prediction framework for learning long-range spatial relationships, which is achieved by using larger receptive fields. This model achieved an accuracy of 87.95 % on MPII dataset [17], which was 6.11 % higher than its closest competitor. HPE, with iterative error feedback [28] introduced a novel self-correcting model that refined its prediction through multiple stages. This approach has also been adopted by the Stacked Hourglass Networks, where the features are down-sampled and up-sampled through multiple stages, to distil relevant features and predict heatmaps [29]. While most feature extraction networks for Human Pose Estimation (HPE) diminish feature resolution during propagation through the model, HRNet [30] preserves a high-resolution representation throughout the entire process, resulting in superior performance relative to existing methods. In the pursuit of a simple but effective pose estimation solution, Simple Baselines [31] for human pose estimation and tracking was introduced. It uses a feature extractor and a stack of deconvolution blocks to upscale the extracted features to a set of heatmaps, one corresponding to each key-point.

Despite these advancements in general-purpose HPE, in-bed pose estimation faces unique challenges such as privacy concerns, varying illumination, body occlusions, and data scarcity. Recent works ([13,32]) demonstrated the effectiveness of deep learning models trained on LWIR images for this application. A recent paper [33] explored Ultra-Wideband (UWB) radar images for detecting sleep pose transitions. Other studies [34] investigated the use of multiple non-visual imaging modalities like depth, LWIR, and pressure maps for in-bed pose estimation, focusing on modality fusion for enhanced privacy and robustness. Another research work [35] proposed a multi-modal conditional variational auto-encoder (MC-VAE) framework using self-supervised learning to reconstruct features from missing modalities, achieving competitive results with single-modality data.

Deep learning models often struggle with domain mismatch, where training data and real-world application data differ significantly. This issue is particularly acute for LWIR images due to the scarcity of annotated data. Domain adaptation techniques are therefore critical to address this gap. A study [36] introduces a two-fold data augmentation technique and self-supervised knowledge distillation to reduce domain discrepancies between uncovered and covered LWIR images, thereby improving the robustness and accuracy of pose estimation models in real-world settings. Although this method demonstrates significant improvements, it employs a teacher-student model without data filtering, introducing errors in training data. Conversely, statistical analysis of images from different domains can offer more reliable and less error-prone results. Additionally, leveraging a large set of *unannotated* data through semi-supervised learning techniques and transfer learning, along with a data filtration process, can significantly enhance model performance. These approaches improve the generalizability of models to new, unseen data, making them more robust in real-world applications.

## 3 Dataset

**Fig. 1:**
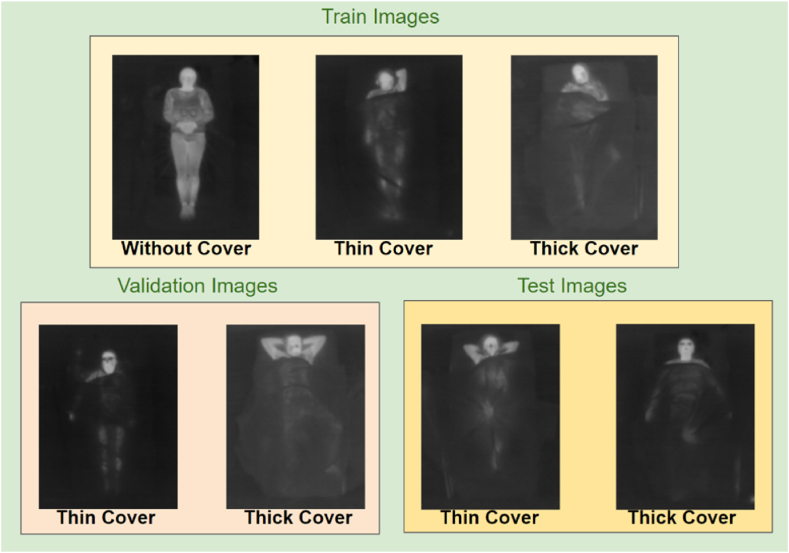
Samples of infrared (IR) images from the SLP dataset depicting subjects in various lying poses, both uncovered and covered with thin or thick blankets, used to train and evaluate the sleeping pose estimation model, with annotations available only for the uncovered images to support the development of the pose estimation framework.

The present work uses a subset of the SLP dataset that contains IR images of subjects lying on the bed with different poses, as shown in Fig 1.

The training dataset contains images of subjects without any blanket or with either a thin or thick blanket on their body. IR emissions are attenuated more in regions where body parts are covered by a blanket. Therefore, pixel intensity is lower in the covered region. There are two types of blankets used - a thin blanket (≈1 mm thickness) and a thick one (≈3 mm thickness). Annotations for 14 body key-points are provided only for images without any blanket. The rest of the training set, with thin or thick blankets, is unannotated. The number of subjects in the training set without any cover, with thin covers and with thick covers, are 30, 25, and 25, respectively. A set of 45 images is provided for each of the subjects, with each image showing a different pose. A summary of the dataset with train, validation and test splits is provided in Table 1.

**Table 1:** SLP dataset details.

Split	Blanket	Subjects	Images	Annotated
Training	None	30	1350	✓
	Thin	25	1125	×
	Thick	25	1125	×
Validation	Thin	2	90	✓
	Thick	2	90	✓
Test	Thin	3	135	✓
	Thick	3	135	✓

All subjects in the validation and test data are covered with either a thin or thick blanket. In the validation set, there are 2 subjects with thin blankets and 2 more subjects with thick blankets. The test set comprises 3 subjects with thin and 3 subjects with thick blankets. Similar to the training data, 45 images are provided for each of the subjects.

## 4 Method

### 4.1 Data preparation

To make up for the lack of annotated covered subjects in the training set, the first step in the data preparation pipeline involved augmenting annotated IR images. By attenuating the pixel intensities of the labeled images, the presence of a blanket could be mimicked. Two different approaches were used for this purpose - the first is based on statistical information of pixel intensities across all training images (covered and uncovered), while the other approach used a GAN model to “paint” a blanket on uncovered images. These approaches are elaborated in the subsequent sections.

For convenience, images of uncovered subjects are denoted as Xu and covered subjects as Xc. Images with thin and thick blankets are denoted as Xc, thin and Xc, thick, respectively. After augmentation, synthetic covered images, Xs, are obtained.

### 4.2 Statistical approach for data augmentation

#### 4.2.1 Statistical attenuation

To mimic the effect of blankets on the IR images, the attenuation coefficient as well as the thickness of the cover need to be known. The attenuated pixel intensities can be computed using Equation −1, where I and I0 refer to attenuated and initial pixel intensities respectively, α is the attenuation coefficient, and x is the thickness of the cover.

(1) $$I=I_{0}e^{-\alpha x}$$

In the SLP dataset, two types of cover were used - thick cover with a thickness of 3 mm and thin cover with a thickness of 1 mm, but the exact material of the fabric, which determines α, was not reported. However, the unlabeled Xc in the training set was used to estimate the attenuation factor, F, the exact value of which is given by rearranging Equation-1 into Equation-2.

(2) $$F=\frac{I}{I_{0}}=e^{-\alpha x}$$

**Fig. 2:**
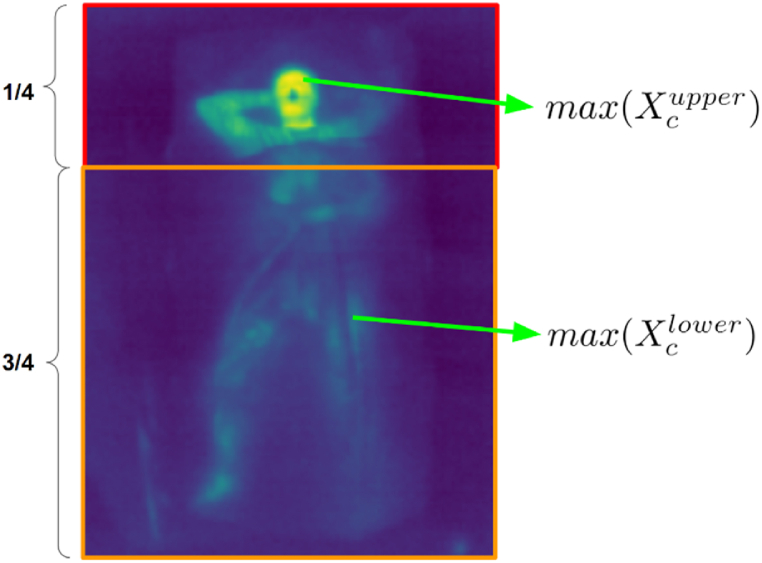
Estimation of the attenuation factor by comparing uncovered and covered regions in IR images, using pixel intensities in the upper region (uncovered) for unattenuated intensities and those in the lower region (covered) for attenuated intensities.

By manually inspecting the dataset, it was found that the region near the subject's head was mostly uncovered in Xc. Conversely, the region below the head (torso and legs) was almost always covered. Thus, the pixel intensities of the head region can be used as a proxy for I0, and pixel intensities near the torso as a proxy for I. This is illustrated in Fig. 2. The upper one-fourth region of the IR images was analyzed, and the maximum pixel intensity, $\max\left(X_{c}^{upper}\right)$, was taken to be the unattenuated intensity of the subject's body. By doing the same for the lower three-fourths of the IR images, an estimate of pixel intensity after attenuation by the cover, $\max\left(X_{c}^{lower}\right)$, was obtained. The attenuation factor, F could then be approximated by Equation-3.

(3) $$F\approx \frac{\max\left(X_{c}^{lower}\right)}{\max\left(X_{c}^{upper}\right)}$$

**Fig. 3:**
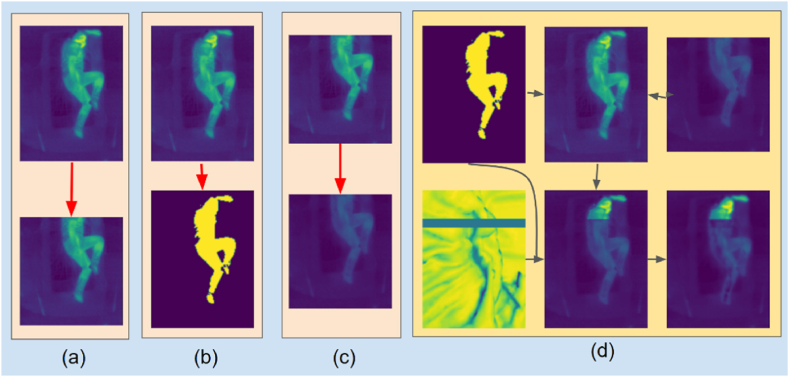
Generation of synthetic covered images. (a) Extracting the lower portion of an uncovered image. (b) Creating a subject mask by applying a threshold. (c) Attenuating the lower portion to simulate a blanket. (d) Combining the mask, attenuated lower portion, and wrinkle pattern to produce the synthetic covered image.

After applying Equation −3 on all images in Xc, a set of estimated attenuation factors was obtained. Next, a portion of the lower three-fourths of each Xu was randomly selected, and the pixels were attenuated with a randomly sampled attenuation factor from the aforementioned set, as illustrated in Fig. 3. The attenuation was limited to be roughly within the area which the subject's body occupied. This was done by first generating a mask for the subject's body using a simple threshold approach (Fig. 3b), and then only attenuating the region within the mask.

Practically, the attenuation due to the blanket is not uniform as it will lay on the subject's body differently in different areas, i.e. the blanket will be wrinkled and fold on itself in different places; this was also the case for Xc. To introduce wrinkles in the synthetic images, the popular and open-source 3D modeling program, Blender was used to running a simple cloth simulation. A bed sheet model was randomly perturbed to get random folding and wrinkle patterns in the cloth. The wrinkled bed sheets were rendered out as grey-scale images and used as a perturbation map for applying attenuation. This process is illustrated in Fig. 3d. Darker areas in the wrinkle map corresponded to folded up cloth, and consequently higher attenuation than usual. Thus, a set of synthetic images, Xs, were generated, using random wrinkle patterns and attenuation factors, on the fly during the training.

#### 4.2.2 Miscellaneous augmentation

**Fig. 4:**
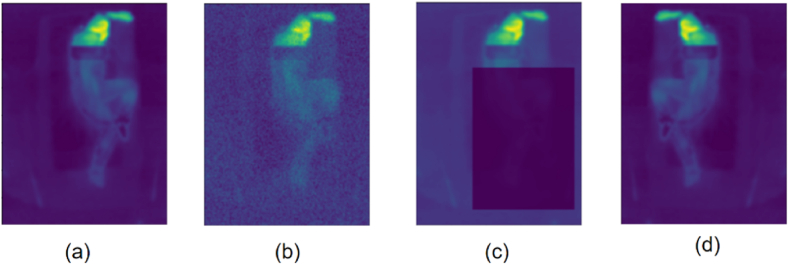
Image augmentations used in the training process: (a) attenuation to simulate a synthetic cover, (b) addition of Gaussian noise to mimic sensor noise, (c) application of heavy attenuation masks to simulate complete obstruction, and (d) horizontal flipping of images to increase variability in the training data.

Besides synthetic attenuation augmentation, other applicable augmentations illustrated in Fig. 4 were also utilized. Noise effects (simulating noise in the camera sensor, flickering lights, dust in the air, etc.) were introduced by adding random noise to the training images. Additionally, complete obstruction was simulated by heavily attenuating random regions in the image. Finally, horizontal flipping was applied, which required altering annotations as left and right limbs would be swapped. During training, the augmentations were enabled and compounded randomly on the fly.

### 4.3 GAN based augmentation

**Figure-5:**
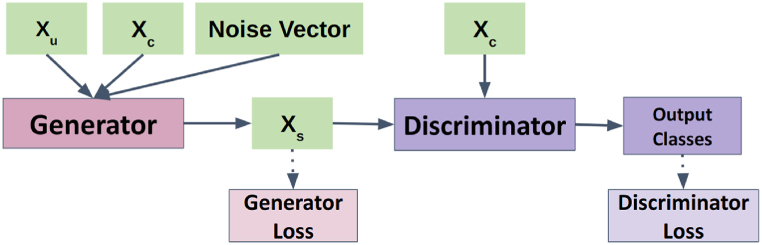
GAN model architecture for synthesizing covered images (Xc), showing the generator taking an uncovered image (Xu), a covered image (Xc) and a noise vector as input to produce a synthetic covered image (Xs), while the discriminator distinguishes between real and synthetic covered images.

In order to create more realistic synthetic images, Generative Adversarial Networks (GANs) was also explored for style transfer [37]. That is, the goal was to transfer the “style” of Xc - which includes the attenuation by the wrinkled covers - onto Xu to synthesize Xs. The GAN architecture (Figure-5) consists of two models working against each other: a generator, synthesizing images, and a discriminator, trying to tell synthetic images apart from real ones.

In this particular application, the generator model would take an example from Xc and Xu, along with a noise vector (to introduce variation) as inputs and generate Xs. The discriminator would then take a set of images from Xs and Xc and try to classify these images. If Xs does not represent Xc accurately enough, the discriminator should be able to classify them easily.

**Figure-6:**
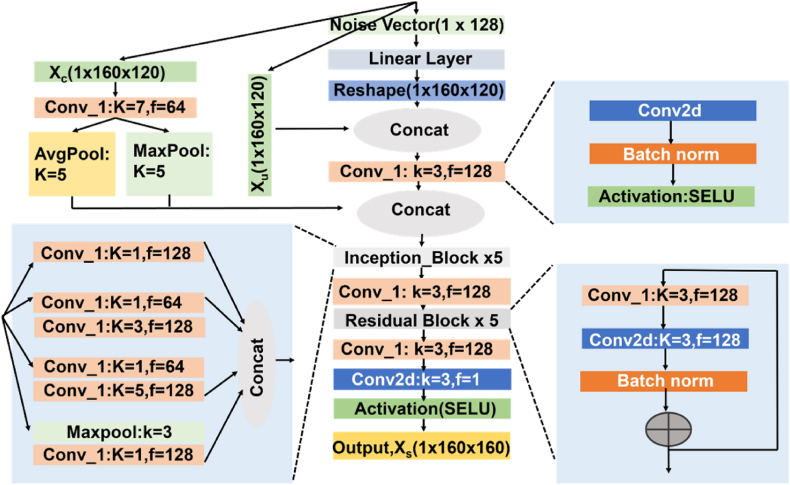
Generator model architecture in the GAN framework, showing the generator taking an uncovered IR image (Xu), a noise vector, and a style reference image (Xc) from the covered dataset to produce a synthetic covered image (Xs). The model uses convolutional layers, inception blocks, and residual blocks to generate realistic covered images that simulate the effect of blankets.

The generator model, G, is shown in Fig. 6. Firstly, a randomly generated noise vector (uniformly distributed between −0.5 and 0.5) is transformed using a linear layer, the output of which is reshaped into a 2D map, N, with the same shape as Xu. N is then concatenated with Xu and encoded using a 3 × 3 convolution layer. An image randomly selected from Xc as the “style reference” is separately encoded using a 7 × 7 convolution layer, and then pooled separately using average pooling and max pooling, both with 5 × 5 kernels and stride of 1. The output of the average pooling and max pooling are concatenated, producing S, which represents the overall pixel intensity distribution in Xc without including fine details in Xc. S is then concatenated with the encoded representation of Xu and N, which go through an inception block [38], a 3 × 3 convolution layer, five residual blocks [39] (shown inset in Figure-6) in and two more 3 × 3 convolutional layers. The output of the final layer is the synthesized image Xs. All convolutional layers use a stride of 1 and apply padding to keep the output shape as the input. Batch Normalization [40] and SELU activation function [41] were used after each convolution, except for the final layer.

The discriminator model, D, is shown in Figure-8. It takes an image, either from Xs or Xc and transforms it using an initial 3 × 3 convolution layer, an inception block, and then six 3 × 3 convolutions. The final output is a 10 × 8 map (one-sixteenth the size of the input). If the input is from Xs (i.e. synthesized by generator), the discriminator is trained to output all zeros in the 10 × 8 map (0). Conversely, if the input is from Xc (i.e. original covered image), the discriminator was trained to produce all ones (1). Like the generator, all convolutions are followed by batch normalization and SELU activation, except the final layer.

**Figure-7:**
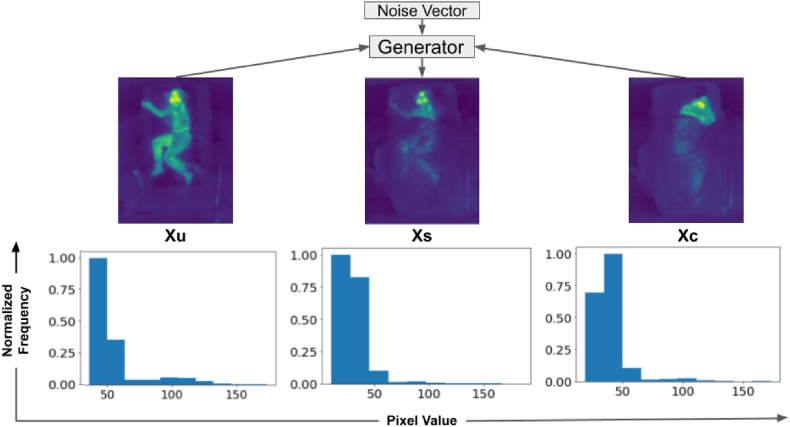
Generator input and output example, showing (a) an uncovered IR image (Xu) used as input, (b) the style reference image (Xc) from the covered dataset, and (c) the synthetic covered image (Xs) generated by the GAN model. (Corresponding normalized distributions of pixel values in the images are given below.)

**Figure-8:**
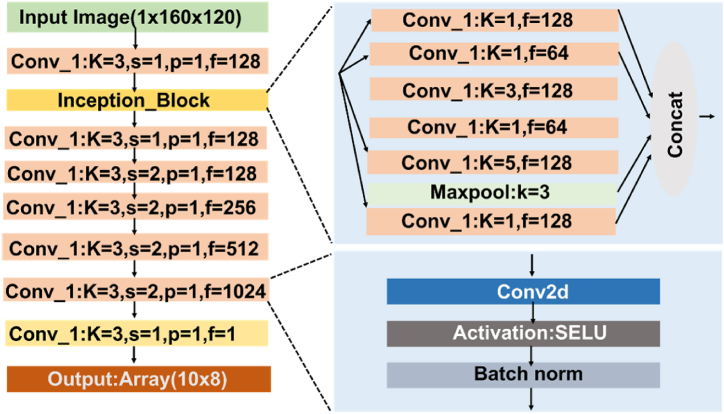
Discriminator model architecture in the GAN framework, where the discriminator processes an image (either synthetic or real) through a series of convolutional layers and an inception block to determine whether the image is real or generated.

### 4.4 Pose estimation network architecture

**Figure-9:**
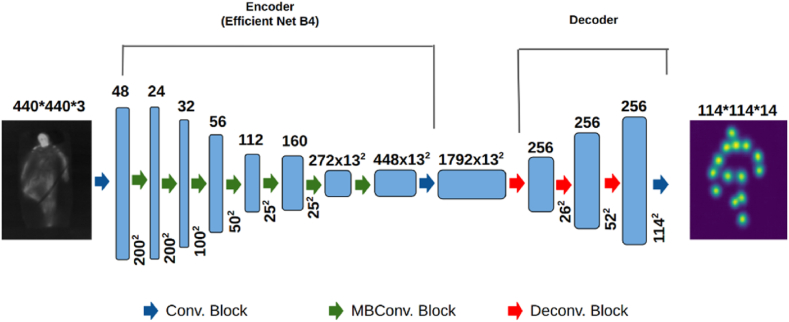
Pose estimation network architecture, consisting of an encoder with an EfficientNetB4 backbone pre-trained on ImageNet, and a decoder with transposed convolutional layers to generate heatmaps for each body key-point.

The architecture adopted is inspired by the work presented in “Simple Baselines for Human Pose Estimation and Tracking” [31]. The architecture consists of an encoder and a decoder. For the encoder, an *EfficientNetB4* [42] backbone initialized with weights from training on the *ImageNet* dataset [43] for image classification is used. The decoder is a series of transposed convolutional layers that upscale the features from the encoder into a set of heatmaps, one corresponding to each body key-point. The network architecture of the proposed model is illustrated in Fig. 9.

The encoder takes an IR image with dimension 440 × 440 × 3 which is scaled up from the original resolution of 160 × 120 × 1 via nearest-neighbor interpolation and cloning channels. In the encoder, the image passes through a convolution block and produces a feature map with dimensions 200 × 200 × 48. This subsequently passes through multiple MB Convolution blocks [42] of the *EfficientNetB4* backbone. The final encoded features have a dimension of 13 × 13 × 1792. The decoder takes this and passes it through 3 deconvolution blocks and generates a feature map with dimension 114 × 114 × 256. Each deconvolution block is followed by batch normalization and ReLU activation. Finally, a convolutional layer with softmax activation produces heatmaps with dimension 114 × 114 × 14. Here, the 14 channels correspond to the 14 annotated body key-points in the SLP dataset. Thus, each 114 × 114 heatmap estimates the pixel-wise probability of a specific key-point.

### 4.5 Semi-supervised training scheme

**Figure-10:**
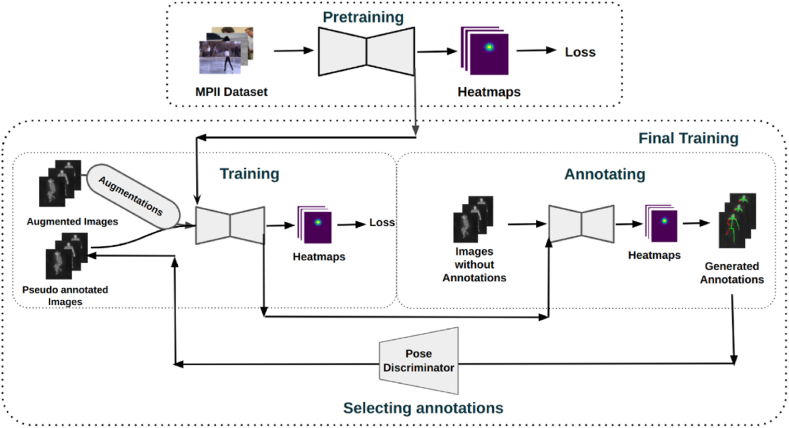
Training scheme for the pose estimation model, consisting of priming on the RGB dataset (MPII) and adapting to the LWIR dataset (SLP) using semi-supervised learning, which includes generating pseudo-labels, filtering with a pose discriminator, and incorporating extensive data augmentation.

The training scheme, illustrated in Figure-10, consists of two phases: priming on RGB datasets, and adapting on the LWIR images of the SLP dataset to utilize all labeled and unlabeled training data through a semi-supervised approach.

#### 4.5.1 Priming on RGB dataset

The proposed model architecture for pose estimation was first primed by training on the MPII dataset [17], which contains RGB images of humans in various poses amidst different activities such as sitting, exercising, dancing, etc. The MPII dataset is a multi-person dataset. It may contain more than one person per image. Since the target domain problem involves only a single person per image, multi-person images in the MPII dataset were split by cropping each individual based on bounding boxes generated from the annotations.

#### 4.5.2 Adapting on LWIR dataset

This phase of the training scheme is an iterative process illustrated in Figure-10. First, all annotated and augmented images from Xu and Xs (described in Section −4.1) were used to train the model. The model with the highest validation score was then used to generate annotation heatmaps hN0 for the unannotated covered images Xc. A separate model, called the Pose Discriminator (see Section-4.6), was used to evaluate the annotations hN0. This model outputs a score between 0.0 and 1.0, where a higher score represents a better annotation. The portion of hN0 that passed with a score higher than the threshold τ was selected and denoted by hN. The images from Xc corresponding to heatmaps hN were included in subsequent training iterations, along with images from Xu and Xs. This process was repeated until no improvement in validation scores was observed.

### 4.6 Pose discriminator model

During the semi-supervised training described in Section-4.5.2, the best model from the previous iteration was used to annotate the unlabeled covered images. Since these pseudo-annotations would not have been perfect, a method to filter those detrimental to training was needed. Initially, a random selection of pseudo-annotated images was included in subsequent training iterations. However, this approach degraded the final model's performance. As illustrated in Figure-12, some predicted annotations were erroneous, resulting in abnormally small limb predictions. To detect and filter these types of bad annotations, a “pose discriminator” was developed. It analyzes the actual image of the pose paired with the original ground truth heatmaps or their deformed versions. Like the pose estimation model, the pose discriminator model was first primed on the MPII dataset, and then on the augmented SLP training dataset.

**Figure-11:**
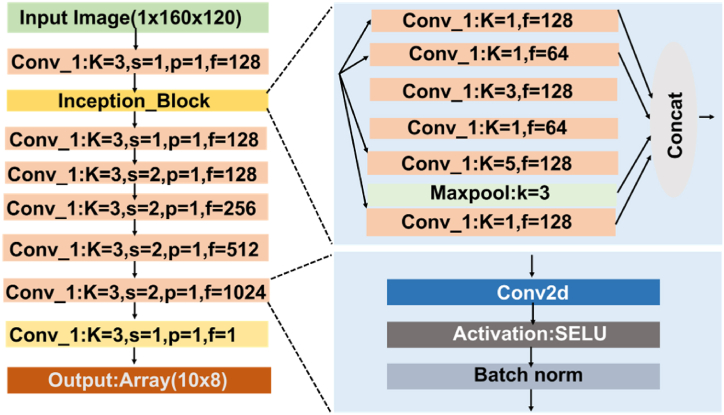
Pose discriminator model architecture, evaluating the quality of pseudo-annotations by analyzing the input image and its corresponding heatmaps via an attention-like mechanism. Higher scores indicate more reliable pseudo-annotations.

The architecture of the pose discriminator model is shown in Fig. 11. A *Resnet101d* [39], initialized with weights from training on the *ImageNet* dataset, is used. The associated heatmaps (original or deformed) for each input image are incorporated in the middle of the network using a "Pose Attention” block. The heatmaps act as spatial attention weights and are multiplied by the feature maps from the previous *Resnet101d* layer. The heatmaps are concatenated with the output for redundancy and are passed through a single 1 × 1 convolution layer. The convolved output has the expected dimensions for being input into the subsequent *Resnet101d* layer. The final output of the *Resnet101d* backbone is transformed by a linear layer, followed by a sigmoid activation. This generates the confidence score between 0 and 1. Binary cross entropy was used as the loss function for this model.

**Fig. 12:**
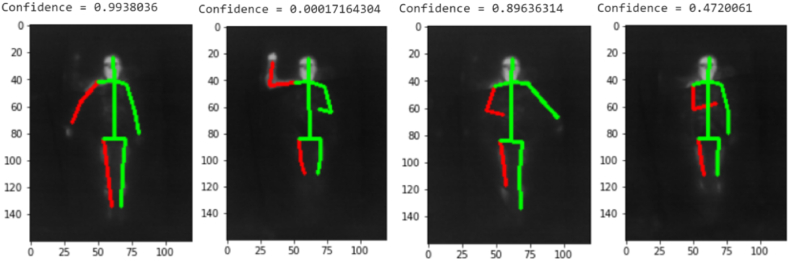
Examples of predictions from the pose discriminator model, showing IR images with predicted key-points. High-scoring annotations accurately match the true pose, while low-scoring annotations contain significant errors, demonstrating the discriminator's effectiveness in filtering out unreliable annotations and improving model performance.

### 4.7 Inference

**Fig. 13:**
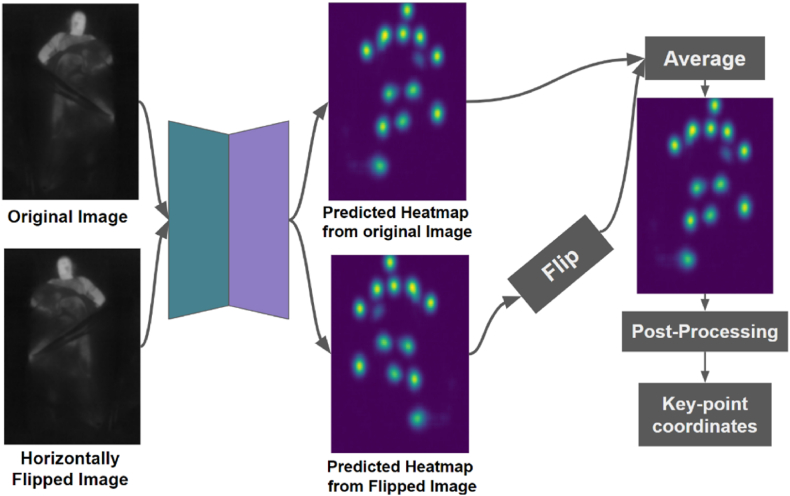
Inference process for the pose estimation model, showing heatmap generation for each body key-point from both original and horizontally flipped images. The heatmaps are averaged, and an iterative refinement process is applied to accurately determine the coordinates of the key-points.

For each input image, 14 heatmaps corresponding to the 14 annotated key-points in the SLP dataset were produced by the pose estimation model. To improve accuracy, each image was decoded twice; first using the original image, and then again with the image horizontally flipped, as shown in Fig 13. The heatmaps from the flipped image were flipped back, and then the arithmetic mean of the two sets of heatmaps was taken as the final output.

To convert these heatmaps into sub-pixel co-ordinates of key-points, an iterative refinement process was applied. The pixel coordinates of the highest value in each heatmap, h, were taken to be the first estimate, C0=(x0,y0). A refined estimate was computed as the center of mass of a d × d window centered on the heatmap at the previous estimate. This process is illustrated in the following algorithm.

h(x,y)←heatmap

$$C_{0}\leftarrow \left(x_{0},y_{0}\right)$$

D←5

N←3

n←0

whilen<Ndo

$$\left(x_{n},y_{n}\right)\leftarrow \left(\left\lfloor x_{n}+0.5\rfloor ,\lfloor y_{n}+0.5\right\rfloor \right)$$

$$S=\sum_{i=-d}^{d}\sum_{j=-d}^{d}h\left(x_{n}+i,y_{n}+j\right)$$

$$x_{n+1}=\frac{1}{S}\sum_{j=-d}^{d}\sum_{j=-d}^{d}h\left(x_{n}+i,y_{n}+j\right)\left(x_{n}+i\right)$$

$$y_{n+1}=\frac{1}{S}\sum_{j=-d}^{d}\sum_{j=-d}^{d}h\left(x_{n}+i,y_{n}+j\right)\left(y_{n}+j\right)$$

$$C_{n+1}\leftarrow \left(x_{n+1},y_{n+1}\right)$$

n←n+1

endwhile

## 5 Experiments

### 5.1 Metrics

Percentage of Correct Key-points (PCKh) [17] was used as the evaluation metric, which is a standard metric for pose estimation problems. PCKh can be evaluated at different thresholds, t, which is a fraction of the head to neck length distance, l. Thus, PCKh@t measures the percentage of predicted key-points that are within t × l distance of the ground truth coordinates. In this paper, values for PCKh@0.5, PCKh@0.2, and the normalized area under the PCKh@t vs. t curve, AUC(PCKh), are presented. The latter is computed using Equation- 4,5 and is a value between 0.0 and 1.0. When expressed as a percentage, AUC%(PCKh) is used.

(4) $$AUC\left(pckh\right)=\frac{1}{0.5}\int_{0}^{0.5}pckh\left(t\right)dt$$

(5) AUC%=100*AUC(pckh)

### 5.2 Training pose estimation model

The two-phase semi-supervised training scheme (Section-4.5) was implemented using the Lightning framework [44] for PyTorch [45], which enabled rapid prototyping. During the priming phase, the model was trained on the processed MPII dataset. As mentioned in Section- 4.5.1, multi-person images in the MPII dataset were converted to single-person images. The annotations provided with the MPII dataset were used to determine the bounding box around each person, which was then cropped and resized to 384 × 384. The dimension of each output heatmap corresponding to this input size is 104 × 104. No augmentation was used for this phase to limit the computational overhead. The model was trained for 25 epochs on the MPII dataset using the AdamW optimizer [46] and a constant learning rate of 0.001. The loss function used is shown in Equation-6; it is the sum of mean squared error (MSE) between each predicted heatmap, hPi, and corresponding ground truth heatmap, hGi, for the 14 key-points.

(6) $$L=\sum_{i=1}^{14}{\left(h_{G_{i}}-h_{P_{i}}\right)}^{2}$$

The model checkpoint with the best validation loss from the priming phase was selected for the next phase of training - adapting on LWIR images from the SLP dataset. During this phase of training, the 160 × 120 LWIR images from the SLP dataset were scaled up to 440 × 440. The corresponding size of generated heatmaps was 112 × 112.

The SLP dataset annotations are provided as pixel co-ordinates of each key-point. To generate ground truth heatmaps for training, a 2D array was created with the same as the input images, and placed 2D Gaussian kernels such that the peak was at the key-point coordinates. The radius of each Gaussian kernel was dynamically reduced from 3 pixels to 1 pixel, over the first 30 epochs. The more diffuse target heatmaps initially helped the model find the general locations of key-points, before optimizing to pinpoint exact locations.

Statistical augmentation techniques described in Section-4.2 were used on-the-fly. For the GAN-based augmentation described in Section-4.3, covered images were synthesized using the best GAN model checkpoint.

The model was trained for up to 100 epochs, with early stopping in place to terminate training when the loss function plateaued or decreased for 10 consecutive epochs. Like the priming phase, the AdamW optimizer was used for this phase, but with a one-cycle learning rate policy [47], with the maximum learning rate set at 0.0009. The same loss function as in the priming phase, shown in Equation-6, was used.

**Fig. 14:**
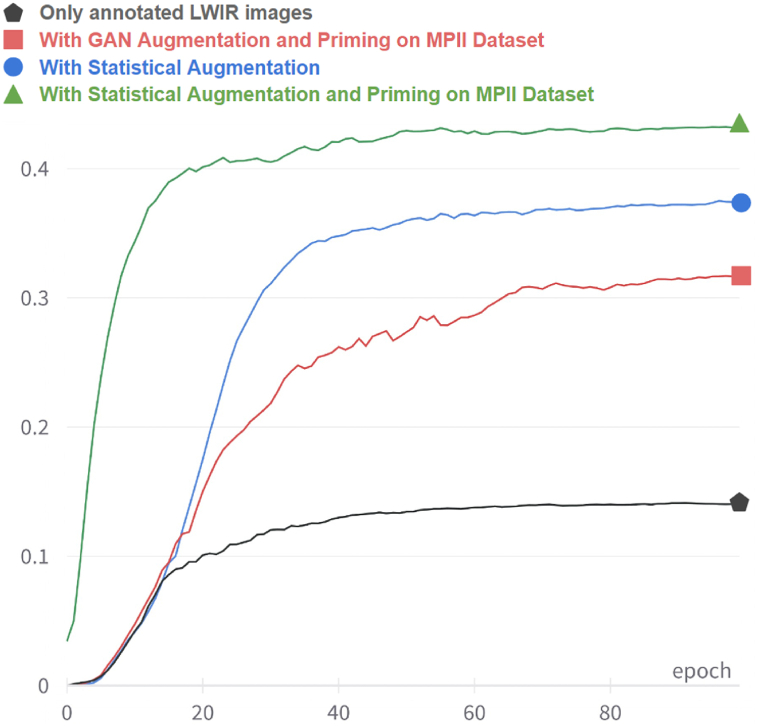
Validation of AUC(PCKh) curves across training epochs, comparing the effects of various aspects of the proposed training scheme, including statistical augmentation, priming on MPII, and semi-supervised training.

The model checkpoint with the best validation loss was used to annotate the covered images in the SLP dataset. Different strategies were then used to sample these pseudo-labeled images. The final model performance using these strategies is reported in Table 4. Initially, all pseudo-annotated images were included, but this led to a less accurate model. Next, 10 % of pseudo-annotated images were randomly selected; while this approach was better than including none or all pseudo-labeled images, there was room for improvement. The pose discriminator model described in Section 4.6 was then used to score the pseudo-annotations and select those that scored above a threshold, τ. Based on validation scores over several training runs, τ = 0.7 was found to be optimal. After selecting the pseudo-annotated images, they were incorporated into the rest of the training data, and the two-phase training was re-run. This entire procedure was repeated for 3 iterations. Fig. 14 shows the validation AUC(PCKh) curve across training epochs with and without various aspects of the training scheme. It is observed that including all the proposed techniques leads to the most accurate model.

### 5.3 Training GAN for synthesizing covered images

To train the GAN model described in Section 4.3, a composite loss function, LG, for the generator was used. It consists of four different terms, as shown in Equation-7. The first term evaluates how well the generator is able to deceive the discriminator, which is the mean squared error between the discriminator's output when given the generator's output and an array of all ones (1). The second term evaluates how similar the synthesized image is to the original uncovered image. The subject's pose in Xs should be the same as in the image used from Xu. To make the generator fit this requirement, the second term takes the MSE between Xs and Xu, but with the latter being scaled by the ratio of mean intensity between the image from Xc used as the style reference in the generator and Xu. The third and fourth loss terms are the MSE between the mean and standard deviation of intensity of Xs and the image from Xc used as the style reference, respectively. These two terms guide the generator to synthesize an image that has a similar pixel intensity distribution as the reference covered image.

$$g1=MSE\left[D\left(X_{s}\right),1\right]$$

$$g2=MSE\left[X_{s},X_{u}\frac{mean\left[X_{c}\right]}{mean\left[Xu\right]}\right]$$

$$g3=MSE\left[mean\left[X_{s}\right],mean\left[X_{c}\right]\right]$$

$$g4=MSE\left[std\left[X_{s}\right],std\left[X_{c}\right]\right]$$

(7) $$L_{G}=g1+g2+g3+g4$$

The discriminator loss, LD, is calculated as shown in Equation-8. It is the arithmetic mean of two terms; the first is the MSE between discriminator outputs when given images from Xc and an array of all ones is computed, and the second is the MSE between the discriminator outputs when given images from Xs and an array of all zeros is computed.

$$d1=MSE\left[D\left(X_{c}\right),1\right]$$

$$d2=MSE\left[D\left(X_{s}\right),0\right]$$

(8) $$L_{D}=0.5\cdot \left(d_{1}+d_{2}\right)$$

**Figure-15:**
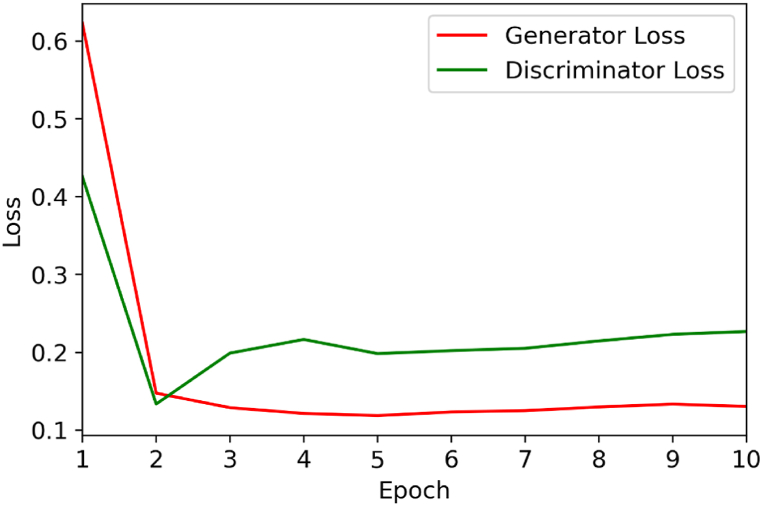
Loss curves of the generator and discriminator during GAN training, showing the training loss profiles for both the generator and discriminator over the epochs.

Random pairs of uncovered and covered images from Xu and Xc, respectively, were created for input into the generator. The generator was trained for 10 epochs independently, that is, without including the g_1_ term in Equation-7. Then, both the generator and discriminator were trained in tandem for 10 more epochs. AdamW was used as the optimizer for both networks with an initial learning rate of 0.001, which was decayed each epoch by a factor of 0.9. The loss profiles of both Generator and Discriminator are shown in Fig. 15.

### 5.4 Experiments with different encoder backbones

**Figure-16:**
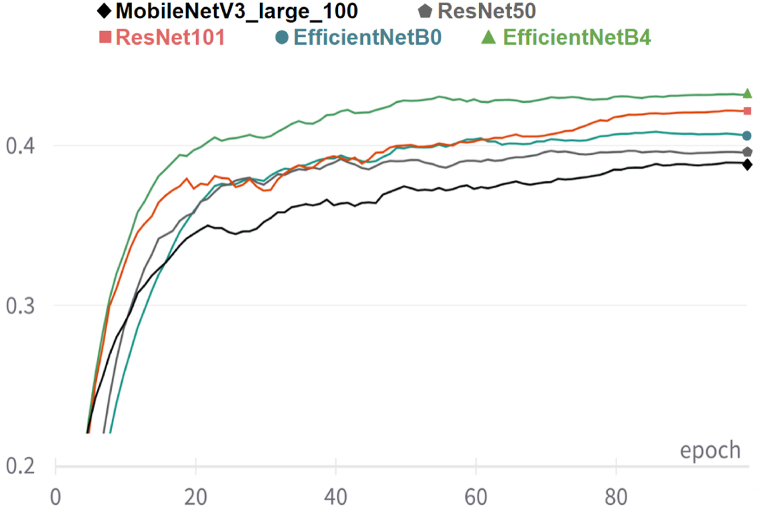
AUC(PCKh) on the SLP validation set using different encoder backbones, comparing the validation AUC(PCKh) curves across training epochs for various encoder backbones, including EfficientNetB0, EfficientNetB4, ResNet50, ResNet101, and MobileNetV3 Large.

In the model architecture proposed in Section-4.4, different encoder architectures and pre-trained weights could be chosen. Architectures and pre-trained weights available in the Torch Image Models (timm) repository [48] were experimented with, including two variants of the *EfficientNet* architecture, two variants of the *ResNet* architecture, and a variant of the *MobileNetV3* architecture. The AUC(PCKh) on the validation set vs. epochs during training is presented in Fig. 16. The *EfficientNet* architectures proved to be more effective, achieving higher AUC(PCKh) than the rest, more quickly. More details are presented in Table 3 in Section-6.

**Table 2:** Evaluation of proposed model and training schemes.

Training Scheme	PCKh @0.5	PCKh @0.2	AUC% (PCKh)
Only annotated LWIR images	33.0	14.1	16.8
+ Statistical Augmentation	74.7	38.3	42.3
+ Priming on MPII Dataset	77.8	43.5	46.0
+ Semi-supervised	**80.1**	**44.7**	**47.5**
GAN Augmentation + Priming on MPII	68.6	31.0	37.0

(Each row beginning with a ‘+’ includes the additions from rows above it. The model used here is the one proposed in Section-4.4, with an EfficientNetB4 backbone.).

**Table 3:** Evaluation of different backbones.

Backbone	PCKh @0.5	PCKh @0.2	AUC% (PCKh)	# Parameters
*MobileNetV3 Large*	75.0	40.0	43.5	9.0 M
*ResNet50*	76.3	42.1	44.9	33.9 M
*ResNet101*	76.9	42.4	45.7	52.9 M
*EfficientNetB0*	77.1	41.2	45.3	11.4 M
*EfficientNetB4*	**77.8**	**43.5**	**46.0**	**27.0 M**

(All models were trained with statistical augmentation and priming on the MPII dataset. Semi-supervised training was not used for these results.).

### 5.5 Experiments with other model architecture

The proposed architecture described in Section-4.4 was compared to two other state-of-the-art architectures - HRNet [30] and Stacked Hourglass [29]. The official implementations of these networks on GitHub were used and included in the training pipeline. The models were trained with the augmentations described in Section-4.2 and were first primed on the MPII dataset as described in Section-4.5.1. The detailed results are presented in Table 1.

## 6 Results

The results from the experiments are summarized in this section. First, Table 2 compares the models trained without and with the various techniques proposed in the previous sections. It can be seen that the model trained solely on annotated (uncovered) images, without any augmentation or priming, performed poorly with an AUC%(PCKh) of 16.8 %. Including the augmentation techniques from Section-4.2, such as synthetic attenuation, random noise, and horizontal flipping, greatly increased the model's performance; the AUC%(PCKh) jumped to 42.3 % with a corresponding large increase in PCKh@0.5. Adding the priming phase of training described in Section-4.5.1 further increased the AUC%(PCKh) to 46.0 %, a relative increase of 8.7 %. By also using the semi-supervised approach of pseudo-labeling covered images and using them for training as described in Section-4.5.2, the highest AUC%(PCKh) of 47.5 % was observed, with a relative increase of 1.5 %. The PCKh@0.5, on the other hand, showed a relative increase of 3.0 %.

Using the synthesized covered images from the GAN described in Section 4.3 caused a reduction in model accuracy, with an AUC%(PCKh) of 37.0 %. Some synthesized samples from the GAN looked realistic and similar to actual LWIR images with covered subjects (see Fig. 7), but when many of them were included with other augmented data, it led to a less optimal solution.

As described in Section-4.4, the encoder in the proposed pose estimation model had an *EfficientNetB4* backbone. Table 3 compares the use of other choices for a backbone. The *EfficientNet* architectures outperformed the *ResNet* architectures. The smallest backbone was the *MobileNetV3 Large* model. It is three times smaller than the *EfficientNetB4* model, yet it performed reasonably well, being about 5.4 % (relative) worse in terms of AUC%(PCKh) than *EfficientNetB4*.

**Figure-17:**
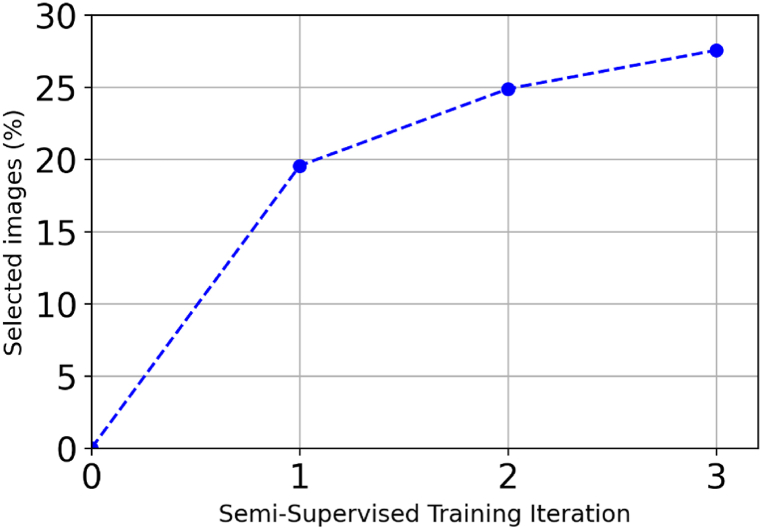
Increasing proportion of high-quality pseudo-annotations selected across iterations by the pose discriminator with τ = 0.7 at each iteration of semi-supervised training, indicating the pose discriminator's effectiveness in improving training data quality and model performance.

In Section 4.6, the ways considered for filtering bad pseudo-annotations during the iterative semi-supervised training were discussed. Namely, three strategies were considered - using all pseudo-annotations, randomly selecting a proportion of them, or using a “pose discriminator” neural network to score pseudo-annotations. The evaluation of these strategies is presented in Table 4. Randomly selecting 10 % of pseudo-annotated images and mixing them into training was superior to mixing in all of them, achieving an AUC%(PCKh) of 41.4 %, a relative improvement of 6.4 %. Using the pose discriminator model was even more effective, raising the AUC%(PCKh) to 45.7, a relative improvement of 10.4 % over random selection. Fig. 17 shows the proportion of images that the pose discriminator selected at every iteration of semi-supervised training. It can be seen that the proportion of images selected increased with each iteration, reaching 27 % of pseudo-annotated images by the end of the third iteration.

The proposed model was compared with other state-of-the-art human pose estimation models, namely Stacked Hourglass and HRNet. The results are presented in Table 5. It is observed that the proposed architecture outperformed both, achieving a relative improvement in AUC%(PCKh) of 4.8 % and 6.0 %, respectively.

**Table 4:** Evaluation of different strategies for choosing pseudo annotated data in semi-supervised training.

Strategy	PCKh @0.5	PCKh @0.2	AUC% (PCKh)
Select all pseudo-annotated images	77.6	28.0	38.9
Randomly Select 10 %	79.0	32.6	41.4
Select with Pose Discriminator (τ = 0.7)	**80.1**	**44.7**	**47.5**

(All models were trained using the EfficientNetB4 backbone, and included statistical augmentation and priming on the MPII dataset.).

**Table 5:** Evaluation of different model architectures.

Model	PCKh @0.5	PCKh @0.2	AUC% (PCKh)	# Parameters
Stacked Hourglass	74.2	41.4	43.9	6.7 M
HRNet	75.6	38.8	43.4	28.5 M
Proposed	**77.8**	**43.5**	**46.0**	**27.0 M**

(All models were trained with statistical augmentation and priming on the MPII dataset. Semi-supervised training was not used for these results.).

The performance of the proposed model was compared with a baseline model trained and evaluated using the same data with domain mismatch, as shown in Table 6. The proposed model outperformed the baseline (Afham et al., 2022) by 5.2 %. It is noteworthy that recent works ([34,35]), which utilized both visible and LWIR modalities, had reported higher accuracy. However, the proposed method in this paper stood out because it used only a small fraction of the labeled data, incorporating the remaining unlabeled data through a semi-supervised training approach. Crucially, the proposed method in this paper exclusively utilized LWIR modality data, highlighting its effectiveness in handling domain mismatch scenarios with limited labeled data.

**Table 6:** Result comparison of methods from different literature.

Method	PCKh@0.5
Afham et al. (2022) [36]	76.13
Proposed Method	**80.1**

(Both models address domain mismatch issues and are trained on the same dataset. The first method employs a semi-supervised approach based on a teacher-student model.).

## 7 Conclusion

In this work, an end-to-end pipeline is introduced for estimating human pose from infrared images, which requires less training data by leveraging domain-specific statistical image augmentation and transfer learning. The statistical augmentation is designed in a way that the model can predict body key-points for an image even when it is taken under adverse vision conditions such as full darkness and/or complete occlusion. The proposed method outperforms the state-of-the-art HPE models by a significant margin. Though this pipeline is designed mainly for in-bed patient monitoring, its application can be extended to a number of applications. The proposed model is robust, being effective even under challenging conditions like darkness and occlusion. Additionally, it is efficient as it utilizes a combination of annotated and unannotated data, reducing the need for extensive labeled datasets. However, there are some drawbacks as well. The semi-supervised and augmentation techniques add complexity to the training process. Furthermore, the model's performance may vary depending on the pose discriminator model's performance. Moreover, GAN augmentation does not perform well, as generating infrared images using GANs is challenging due to the complexity of their structure.

Future research could explore using the statistical augmentation approach and pose discriminator for various non-in-bed pose estimation scenarios from infrared images and other modalities. This could help the computer vision community develop more sophisticated methods in this field, which would be extremely essential, especially due to the disproportionate ratio of health workers to patients during a pandemic or outbreak of any community-wide disease.

## Data availability

The datasets generated and analyzed during the current study are not publicly available. However, data are available from the corresponding author upon reasonable request and subject to necessary permissions or consents (IEEE Video and Image Processing Cup 2021 at ICIP 2021). This arrangement ensures that the data supporting the findings of this study are preserved and accessible under conditions that safeguard privacy and ethical considerations.

## CRediT authorship contribution statement

**Shahriar Kabir Nahin:** Conceptualization, Formal analysis, Methodology, Writing – original draft, Writing – review & editing. **Sanjay Acharjee:** Methodology, Validation, Writing – original draft. **Sawradip Saha:** Methodology, Validation, Writing – original draft. **Aurick Das:** Writing – original draft, Writing – review & editing. **Shahruk Hossain:** Methodology, Supervision, Writing – original draft, Writing – review & editing. **Mohammad Ariful Haque:** Supervision, Writing – original draft, Writing – review & editing.

## Declaration of competing interest

The authors declare that they have no known competing financial interests or personal relationships that could have appeared to influence the work reported in this paper.
